# Occult Breast Cancer Diagnosed from a Solitary Scalp Metastasis without Axillary Lymph Node Involvement: A Case Report

**DOI:** 10.70352/scrj.cr.25-0553

**Published:** 2025-12-09

**Authors:** Tomoka Ushio, Saeko Teraoka, Yoshiya Horimoto, Hiroki Kusama, Yuji Sugiyama, Takashi Ishikawa, Masahiko Kuroda, Shingo Ikeda

**Affiliations:** 1 Department of Thoracic and Breast Surgery, Kohsei Chuo General Hospital, Tokyo, Japan; 2 Department of Breast Surgery and Oncology, Tokyo Medical University, Tokyo, Japan; 3 Department of Gastrointestinal Surgery, Kohsei Chuo General Hospital, Tokyo, Japan; 4 Department of Pathology, Kohsei Chuo General Hospital, Tokyo, Japan; 5 Department of Molecular Pathology, Tokyo Medical University, Tokyo, Japan

**Keywords:** breast neoplasms, occult primary neoplasms, scalp neoplasms/secondary, neoplasm metastasis

## Abstract

**INTRODUCTION:**

Occult breast cancer (OBC) is defined as metastatic breast carcinoma without detectable breast lesions on clinical or imaging evaluation. Most cases are diagnosed from axillary lymph node metastasis (ALNM), while presentation from other sites is rare.

**CASE PRESENTATION:**

A 49-year-old woman with prior gastric cancer developed a 20-mm scalp mass, which was surgically excised. Pathology and immunohistochemistry confirmed metastatic breast carcinoma (hormone receptor-positive and HER2-negative). No breast or axillary lesions were detected by imaging, including PET-CT scan. She was diagnosed with OBC without ALNM and started endocrine therapy with anastrozole.

**CONCLUSIONS:**

We report a rare case of OBC diagnosed based on a solitary scalp metastasis without ALNM. OBC without ALNM appears to favor luminal type and distinct metastatic sites, but further cases are needed to establish treatment strategies.

## Abbreviations

ALN
: axillary lymph node
ALNM
: axillary lymph node metastasis
CDK4/6
: cyclin-dependent kinase 4 and 6
CDX2
: caudal-type homeobox 2
CK7
: cytokeratin 7
CK20
: cytokeratin 20
ER
: estrogen receptor
GATA3
: GATA binding protein 3
GCDFP-15
: gross cystic disease fluid protein 15
HER2
: human epidermal growth factor receptor 2
PgR
: progesterone receptor
MAC
: microcystic adnexal carcinoma
OBC
: occult breast cancer
SEER
: Surveillance, Epidemiology, and End Results

## INTRODUCTION

Carcinoma of unknown primary is defined as a malignant tumor that is confirmed pathologically at metastatic sites, despite no obvious primary tumor being identified on clinical or radiological evaluation. In particular, when no primary lesion is detected in the breast by clinical or imaging studies and the diagnosis is made as metastatic breast carcinoma, the condition is defined as OBC, which is rare, accounting for approximately 0.1%–0.8% of all breast cancers.^1–3)^ OBC is usually diagnosed on the basis of ALNM, whereas cases diagnosed from metastatic sites other than the ALNs are very rare.^3)^ In any case of OBC, identification of the primary site through detailed pathological evaluation, including immunohistochemistry, is critically important for subsequent therapeutic decision-making.

Here, we report a rare case of OBC diagnosed from a scalp skin mass, together with a review of the relevant literature.

## CASE PRESENTATION

In 20XX–3, a 49-year-old postmenopausal woman underwent surgery for gastric cancer (goblet cell adenocarcinoma, pT1a(M)N0M0, Stage IA) at our hospital. In 20XX, a 20-mm scalp skin mass appeared, for which she consulted a plastic surgeon at another hospital. Surgical excision of the scalp tumor was performed, and the pathological diagnosis was adenocarcinoma. As cutaneous metastasis from gastric cancer was suspected, she was referred to our hospital for further evaluation.

**Fig. 1:**
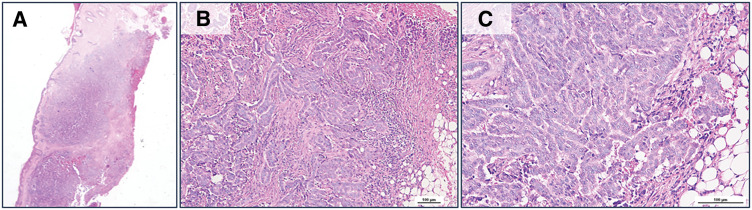
Histopathological images of the skin tumor. Hematoxylin and eosin-stained sections at (**A**) lower power, (**B**) ×100, and (**C**) ×200 magnification.

**Fig. 2:**
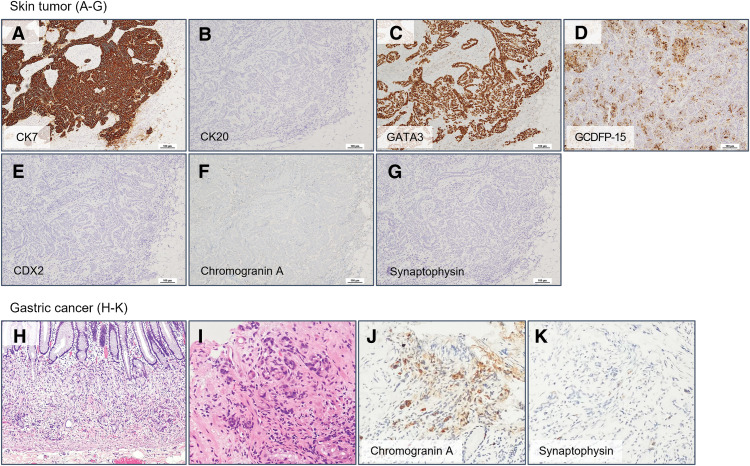
Immunohistochemical findings for differential diagnosis. Immunohistochemical findings of the skin tumor: (**A**) CK7, (**B**) CK20, (**C**) GATA3, (**D**) GCDFP-15, (**E**) CDX2, (**F**) chromogranin A, and (**G**) synaptophysin. Histopathological and immunohistochemical features of the previous gastric cancer: (**H**, **I**) hematoxylin and eosin staining, (**J**) chromogranin A, and (**K**) synaptophysin. CDX2, caudal-type homeobox 2; CK7, cytokeratin 7; CK20, cytokeratin 20; GATA3, GATA binding protein 3; GCDFP-15, gross cystic disease fluid protein 15

**Fig. 3:**
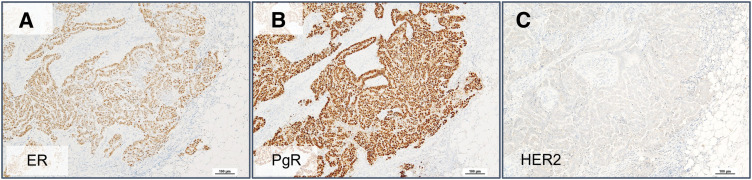
Immunohistochemical staining of ER, PgR, and HER2 for the skin tumor. (**A**) ER, (**B**) PgR, and (**C**) HER2. ER, estrogen receptor; HER2, human epidermal growth factor receptor 2; PgR, progesterone receptor

Histopathological review of the specimen and additional immunohistochemical staining were performed at our institution. The findings, together with those of the previous gastric cancer, are shown in **Figs. 1** and **2**. Small atypical cells were densely proliferating within the dermis, showing a tendency toward tubular formation. This histological feature was not similar to that of the previous gastric cancer (goblet cell adenocarcinoma). Immunohistochemical analysis revealed that the skin tumor was positive for CK7, GATA3, and GCDFP-15, which are characteristic markers of breast carcinoma, and negative for CK20. In addition, CDX2, an intestinal differentiation marker, was negative. The previous gastric cancer, on the other hand, was positive for the neuroendocrine markers chromogranin A and synaptophysin, whereas the present tumor was negative for both. Although there was no apparent continuity between the tumor and adjacent adnexal structures, the possibility of a primary cutaneous adnexal carcinoma still needed to be considered. First, MAC was considered in the differential diagnosis.^4)^ However, no benign adnexal tumor such as syringoma, which often coexists with this neoplasm, was observed around the lesion, and MAC is generally negative for GCDFP-15, which contrasts with the present tumor. Apocrine carcinoma was also considered, but the tumor did not show characteristic features of apocrine differentiation, such as abundant eosinophilic granular cytoplasm. Based on these findings, the lesion was considered most likely to represent metastatic breast cancer. As the phenotype of metastatic breast carcinoma, ER (90%) and PgR (100%) were positive, and HER2 was negative (score 0) (**Fig. 3**). Nuclear grade and histological grade were both Grade 1 and Ki67 labeling index was 15%.

**Fig. 4:**
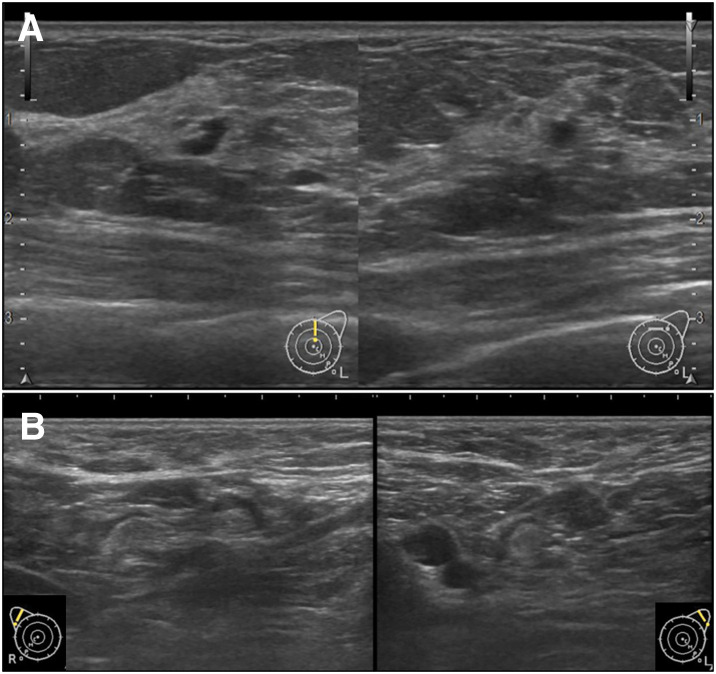
Ultrasound findings of the breast and axillary lymph nodes. Ultrasound findings of the (**A**) left breast and (**B**) bilateral axillary lymph nodes are shown.

**Fig. 5:**
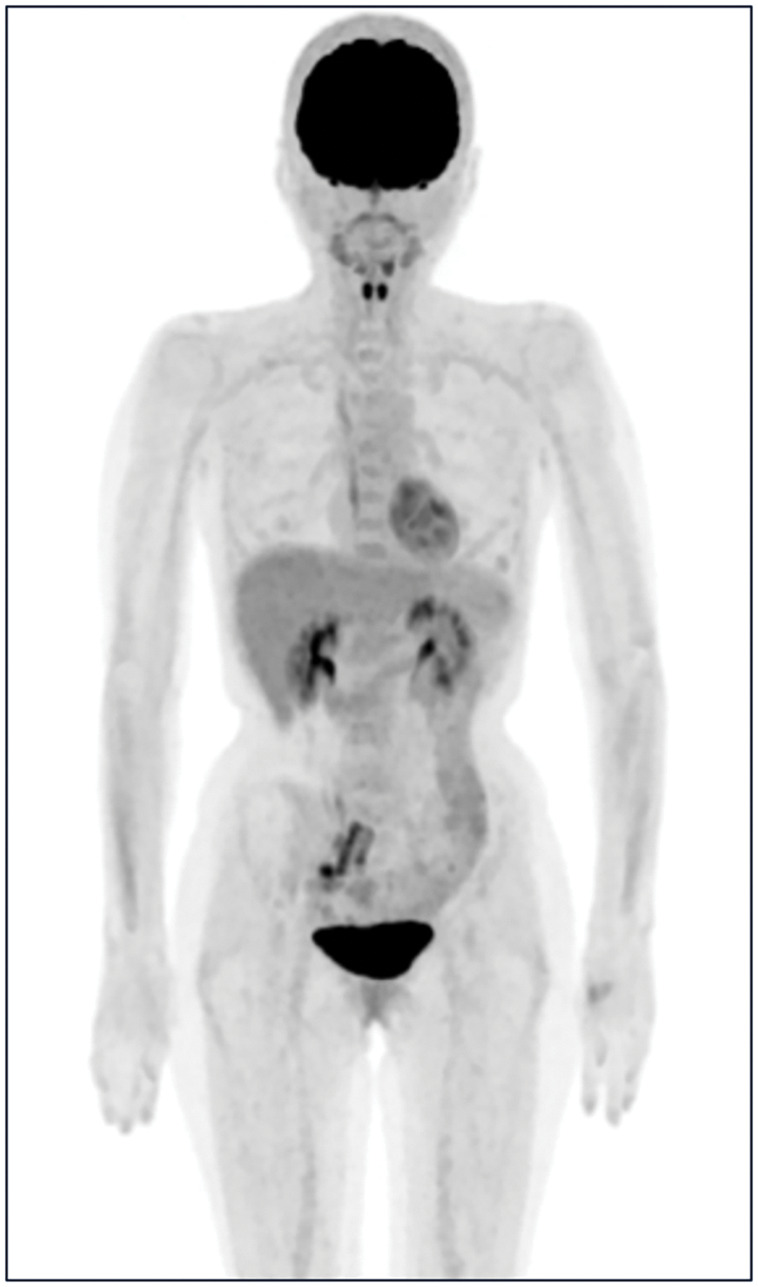
PET-CT findings. The results of the PET-CT performed after referral to our department are shown.

Given the pathological suspicion of metastatic breast carcinoma, she was referred to our department for detailed breast examination. Physical examination revealed no palpable masses in the breast or enlarged ALNs. Mammogram and contrast-enhanced breast MRI showed no significant findings. Breast ultrasound demonstrated a 4-mm cystic lesion in the left breast (**Fig. 4**). Although the lesion was considered benign on imaging, a core needle biopsy was performed for confirmation, given the clinical course, and revealed fibrocystic disease, leading to a diagnosis of mastopathy. No axillary lymphadenopathy was detected on breast ultrasonography. PET-CT scan revealed no abnormal uptake in the scalp, breasts, axillary lymph nodes, or other sites (**Fig. 5**). Laboratory findings were normal, and tumor markers (CA15-3 and CEA) were not elevated.

Based on these results, the patient was diagnosed with OBC without ALNM, and treatment with anastrozole was initiated. She has since been followed up with breast imaging every 3 months, continuing regular ultrasound examinations. Annual mammography is also planned.

## DISCUSSION

On searching the PubMed for cases of OBC diagnosed with initial metastatic sites other than the ALNs, we found that, to the best of our knowledge, no case series compiling multiple cases has been reported, and only 6 individual case reports were identified.^5–10)^ The characteristics of these 6 cases, together with the present case, are summarized in **Table 1**, which highlights that OBC without ALNM tends to present with distinctive metastatic patterns. In the reported cases, either GATA3 or GCDFP-15, or both, were used as breast-specific markers. Notably, 4 cases, including ours, were diagnosed as solitary scalp metastases, which was clearly predominant. Interestingly, Schulman et al. retrospectively analyzed 1984 cases of cutaneous metastases from several cancers and compared the metastatic sites. They demonstrated that, when adjusted for skin surface area, the frequency of metastases was highest in the head and neck region.^11)^ The authors suggested that differences in the local immune environment, such as the high density of regulatory T cell infiltration in the skin of the head and neck, may partly explain this finding, although the precise mechanism remains unclear. Another notable feature of OBC without ALNM was the predominance of the luminal subtype. In contrast, in typical OBC with ALN involvement, hormone receptor positivity has been reported to be slightly lower than in general breast cancer, at just over 60%.^2)^ Therefore, higher hormone sensitivity may represent a distinct feature of OBC without ALNM.

**Table 1:** Reported cases of occult breast cancer without axillary lymph node metastasis

Author (year)	Age/sex	Location	Solitary/multiple	Breast-specific markers	ER and HER2 status	Initial treatment	Outcome
Costa et al.^5)^ (2017)	66 F	Skin (scalp)	Solitary	GATA3	ER^+^, HER2^−^	ANA	N.A.
Alizadeh et al.^6)^ (2018)	44 F	Skin (scalp)	Solitary	GCDFP-15	ER^+^, HER2^−^	EC, DTX, RT^*^, TAM	Alive at 3 years
Barbieri et al.^7)^ (2020)	52 F	Skin (scalp)	Solitary	GATA3, GCDFP-15	ER^+^, HER2^−^	None^**^	Bone and LN met in 28 months
Our case (2025)	49 F	Skin (scalp)	Solitary	GATA3, GCDFP-15	ER^+^, HER2^−^	ANA	Alive at 3 months
Liu et al.^8)^ (2017)	58 F	Bone marrow	Solitary	GCDFP-15	Unknown	None (BSC)	Alive at 1 year
Yamaguchi et al.^9)^ (2020)	59 F	Bone marrow	Solitary	N.A.	ER^+^, HER2^−^	LET, RT^***^, Dmab	Alive at 3.5 years
Neal et al.^10)^ (2009)	53 F	Stomach	Solitary	N.A.	ER^+^, HER2^−^	ANA	N.A.

^*^For scalp.

^**^Patient refused any treatment.

^***^For bone.

ANA, anastrozole; BSC, best supportive care; Dmab, denosumab; DTX, docetaxel; EC, epirubicin plus cyclophosphamide; ER, estrogen receptor; GATA3, GATA binding protein 3; GCDFP-15, gross cystic disease fluid protein 15; HER2, human epidermal growth factor receptor 2; LET, letrozole; LN, lymph node; met, metastasis; N.A., not available; RT, radiation therapy; TAM, tamoxifen

Analyses using the SEER database have reported that the prognosis of OBC with ALNM is comparable to, or slightly better than, that of primary breast cancer at the same stage.^12,13)^ In contrast, considering that OBC without ALNM, including the present case, typically presents as a solitary lesion and is frequently of the luminal subtype, its prognosis is presumed to be even more favorable. Nevertheless, there is currently no solid evidence regarding the prognosis of OBC without ALNM, and further accumulation of cases is warranted.

According to the Breast Cancer Clinical Practice Guidelines 2022 issued by the Japan Breast Cancer Society, the treatment strategy for OBC is described as being equivalent to that for primary breast cancer with ALNM. However, this recommendation is intended for OBC with ALNM, and no specific comments are provided regarding OBC without ALNM.^14)^ The main difference in treatment strategy between OBC with and without ALNM lies in whether surgical and radiotherapeutic interventions for the axilla and breast are included. In the present case, no lesions were detected in either breast or the axillary lymph nodes on imaging. Therefore, we considered that surgical or radiotherapeutic intervention targeting both sides would likely represent overtreatment in this case. As for systemic therapy, although combination therapy with a CDK4/6 inhibitor and endocrine therapy might generally be considered appropriate for metastatic breast cancer, no visceral metastases were identified in this case, and no measurable target lesions were present. Considering the potential adverse effects such as myelosuppression and the financial burden, the additional benefit of intensive systemic therapy was regarded as limited. Therefore, endocrine therapy alone was selected. CDK4/6 inhibitor therapy will likely be incorporated in future second-line treatment. In any case, as no solid evidence has been established regarding the optimal treatment intensity or duration of endocrine therapy in OBC, treatment decisions must inevitably be individualized according to each patient’s condition.

In our investigation, OBC without ALNM was found to metastasize to characteristic organs. For example, while skin metastases may be amenable to local treatment including surgical excision, bone marrow metastases require systemic therapy. Thus, treatment strategies should be prioritized according to the metastatic site. Further accumulation of cases will be essential to establish truly individualized treatment approaches.

In this case, based on histopathological and immunohistochemical findings, we concluded that the skin tumor was most likely a metastasis from breast carcinoma. However, in cases such as this, differentiation from primary cutaneous adenocarcinoma is not always straightforward. There is considerable overlap in the expression of immunohistochemical markers between cutaneous adnexal adenocarcinomas and metastatic breast carcinoma, and no absolute marker exists to clearly distinguish the two. In the present case, the tumor lacked apparent continuity with adjacent adnexal structures, and breast-associated markers such as GATA3 were positive, suggesting a mammary origin. Nevertheless, a primary cutaneous tumor cannot be completely excluded. It is important to recognize the limitations of such differential diagnosis and to make careful judgments.

## CONCLUSIONS

We report a rare case of OBC diagnosed from a scalp skin mass without ALNM. Our case suggests that OBC without ALNM may have characteristic metastatic sites and is frequently of the luminal type, but further accumulation of cases is warranted to establish individualized treatment strategies.
